# Comparative Effectiveness of Long-Acting Lipoglycopeptides vs Standard-of-Care Antibiotics in Serious Bacterial Infections

**DOI:** 10.1001/jamanetworkopen.2025.11641

**Published:** 2025-05-21

**Authors:** David Goodman-Meza, Robert E. Weiss, Michelle L. Poimboeuf, Jeffrey Feng, Tara Vijayan, Marianne Martinello, Gail V. Matthews, Gregory J. Dore

**Affiliations:** 1 Kirby Institute, University of New South Wales Sydney, Sydney, Australia; 2 Division of Infectious Diseases, David Geffen School of Medicine at UCLA (University of California, Los Angeles), Los Angeles; 3 UCLA Center for HIV Identification, Prevention, and Treatment Services, Los Angeles; 4 Department of Biostatistics, Fielding School of Public Health, UCLA, Los Angeles; 5 Medical Imaging Informatics Group, Department of Radiological Sciences, UCLA, Los Angeles

## Abstract

**Importance:**

Serious bacterial infections such as bacteremia, endocarditis, osteomyelitis, and septic arthritis typically require prolonged intravenous antibiotics. Long-acting lipoglycopeptides (laLGPs), such as dalbavancin and oritavancin, offer extended treatment intervals for gram-positive infections that may benefit populations with barriers to traditional treatment, including persons who use drugs (PWUD individuals).

**Objective:**

To assess the effectiveness of laLGPs in managing serious bacterial infections in both PWUD and non-PWUD populations compared with standard-of-care (SOC) antibiotics.

**Design, Setting, and Participants:**

This comparative effectiveness study using a target trial emulation framework included data extracted from the US Cerner Real World Data platform. Individuals hospitalized and discharged for serious bacterial infections between October 1, 2015, and October 1, 2022, were included in the analysis. Data were analyzed from July 7, 2023, to February 28, 2025.

**Intervention:**

Receipt of an laLGP (dalbavancin or oritavancin) vs SOC antibiotics.

**Main Outcome and Measures:**

The primary outcome measure was a composite of readmission, emergency department visit, and inpatient death or discharge to hospice within 90 days post discharge from the index admission. Analyses were stratified by PWUD and non-PWUD status. Clone censor weighting was used to emulate a per-protocol analysis. Hazard ratios (HR) of time to the composite event and 95% CIs were calculated using bootstrapping.

**Results:**

Among 42 067 included individuals, median age was 61 (IQR, 47-73) years, 24 704 were male (58.7%), and 5047 (12.0%) were classified as PWUD. laLGPs were prescribed in 825 individuals (2.0%). There was no statistically significant difference in the composite outcome between the laLGP and SOC groups in both the PWUD (HR, 1.01; 95% CI, 0.88-1.13) and non-PWUD (HR, 0.93; 95% CI, 0.86-1.00) participants.

**Conclusions and Relevance:**

In this study of laLGPs vs SOC, findings suggested that laLGPs were effective as step-down treatment of serious gram-positive bacterial infections, offering comparable outcomes to those of SOC antibiotics in PWUD and non-PWUD individuals. Clinicians may consider laLGPs as alternative step-down options to SOC antibiotics for the treatment of serious gram-positive bacterial infections.

## Key Points

**Question:**

What is the comparative effectiveness of long-acting lipoglycopeptides (laLGPs) vs standard-of-care antibiotics as a step-down treatment of serious gram-positive bacterial infections?

**Findings:**

In this comparative effectiveness study using target trial emulation that included 42 067 adults in the US, laLGPs showed similar results in a composite outcome of readmission, emergency department visit, or inpatient death within 90 days post discharge compared with standard-of-care antibiotics.

**Meaning:**

These findings suggest that laLGPs are an effective alternative to standard-of-care antibiotics for the step-down treatment of serious gram-positive bacterial infections.

## Teaser

This comparative effectiveness study evaluates the off-label use of long-acting lipoglycopeptides compared with standard-of-care antibiotics for serious bacterial infections among individuals who do and do not use drugs.

## Introduction

Serious bacterial infections, such as bloodstream infections (BSIs), endocarditis, septic arthritis, osteomyelitis, and pyomyositis, have increased in the US.^1,2,3,4^ Serious bacterial infections are often treated with intravenous or oral antibiotics^5^ for at least 4 weeks. Intravenous antibiotics can be administered as outpatient parenteral antibiotic therapy (OPAT) through short-term vascular catheters, though frequent laboratory monitoring and challenges with adherence may be prohibitive for many patients.^6^ Clinicians face significant barriers in offering OPAT to people who use drugs (PWUD),^7,8,9,10^ including the inability to get home health care agencies to care for such patients due to stigma. The lack of OPAT for PWUD individuals results in prolonged hospitalizations, higher rates of patient-directed discharge, premature treatment discontinuation, and mortality.^11,12^ Long-acting lipoglycopeptides (laLGPs), such as dalbavancin and oritavancin, offer promising new treatment options for both PWUD and non-PWUD groups with these infections.

Dalbavancin and oritavancin, with half-lives of 346 and 393 hours, respectively, show promise in treating serious bacterial infections.^13,14,15^ Therapeutic concentrations of laLGPs may remain above minimum inhibitory concentrations for many gram-positive organisms in serum and deep tissues for up to 8 weeks.^16^ Although currently approved only for the treatment of acute bacterial skin and skin structure infections, several case series highlight the use of laLGPs for serious bacterial infections.^17,18,19,20,21,22,23,24^ Limitations of the published evidence relate primarily to study design, including small sample size, single center, lack of a comparison group, and lack of randomization.

laLGPs have the potential to serve as step-down treatments and alternatives to daily inpatient intravenous antibiotics, OPAT, or oral antibiotics.^17,25^ Our objective was to evaluate the off-label use of laLGPs for serious bacterial infections among PWUD and non-PWUD groups in a large clinical database comparing laLGPs with other treatment regimens. We hypothesized that individuals receiving laLGPs would have similar or better outcomes to those receiving standard of care (SOC) antibiotics.

## Methods

### Specification of the Target Trial

We specified a randomized pragmatic target trial to evaluate the comparative efficacy of laLGPs compared with SOC antibiotics in adults hospitalized with a serious bacterial infection who survived until discharge in reducing postdischarge outcomes at 90 days. eTable 1 in Supplement 1 summarizes the protocol components. We emulated the target trial using observational data from a US deidentified big data source of multicenter electronic health records from more than 100 million patients and 1.5 billion encounters (Cerner Real World Data platform).^26^ We adhered to Reporting of Studies Conducted Using Observational Routinely Collected Data (RECORD) guidelines.^27^ This study was deemed exempt from ethics review by the institutional review board at UCLA, given the deidentified nature of the database. The current analysis was performed from July 7, 2023, to February 28, 2025.

### Study Population

We included individuals in the emulated trial that met the following criteria: (1) 18 years or older; (2) serious bacterial infections defined as a diagnosis of endocarditis, BSI, osteomyelitis, or septic arthritis; (3) time of diagnosis between October 1, 2015, and October 1, 2022; (4) diagnosis associated with an emergency department visit or hospital admission; and (5) receipt of at least 7 days of antibiotics. We set 7 days as the minimum duration for antibiotic treatment to increase the likelihood that the included cases represented clinically relevant presentations of the specified infections. We used the first episode of the inclusion diagnosis in the analysis as the index encounter, and patients were only included once in the analysis. We chose the date range beginning October 1, 2015, as this was the earliest date laLGPs were prescribed, and ending October 1, 2022, to allow for at least 90 days of follow-up for all individuals.

We excluded individuals with (1) a concomitant central nervous system infection; (2) endocarditis requiring early surgical intervention (<10 days from initial diagnosis date); (3) presence of a prosthetic heart valve; (4) presence of a cardiac device (implantable cardiac defibrillator, pacemaker); (5) presence of a transplanted organ (kidney, heart, lung, liver, pancreas, bone marrow); (6) end-stage kidney disease or dialysis; (7) end-stage liver disease; (8) death during the index hospitalization; (9) receipt of terminal antibiotics not typical for a gram-positive infection; and (10) receipt of laLGP prior to the day of discharge. To identify diagnostic conditions and procedures, we searched *International Classification of Diseases, Ninth Revision* (*ICD-9*), *International Statistical Classification of Diseases, Tenth Revision* (*ICD-10*), Systematized Nomenclature of Medicine (SNOMED) Clinical Terms, and *Current Procedural Terminology* codes. Diagnostic and procedure codes and timeframes used to identify these criteria are available in eTables 2 to 4 in Supplement 1. We used dates for inclusion and exclusion criteria to ensure that the criteria were considered prior to the prescription of laLGPs.

### Exposures

Our primary exposure variable was the prescription or no prescription of an laLGP (dalbavancin or oritavanacin) for a serious bacterial infection. We considered individuals who were not prescribed an laLGP to be in the SOC group. Participants in the SOC group had to receive an antibiotic typical for a gram-positive infection as their end-of-therapy antibiotics. We defined antibiotics typical for use in gram-positive infection as monotherapy with vancomycin hydrochloride, daptomycin, oxacillin sodium, nafcillin sodium, dicloxacillin sodium, flucloxacillin, cephalexin, cefazolin sodium, cefadroxil, ceftaroline fosamil, trimethoprim-sulfamethoxazole, doxycycline, minocycline hydrochloride, linezolid, or tedizolid phosphate. Combinations with rifampin were also included. We used receipt of end-of-therapy antibiotics typical of gram-positive infections as a proxy for gram-positive infections due to the absence of microbiological data in the database.

Time zero was defined as the date of discharge. We set a grace period of 10 days from the date of discharge for individuals to receive the laLGP. We set the 10-day grace period as individuals may receive laLGPs outside of the initial hospitalization (eg, at outpatient infusion centers or skilled nursing facilities) for billing and reimbursement purposes.^28,29^

### Stratums

We used previously published codes from the Centers for Disease Control and Prevention and phenotyping libraries as well as keywords related to substance use to classify individuals as PWUD or non-PWUD.^30,31,32,33,34^ We classified individuals as PWUD if they had an *ICD-9* or *ICD-10* code or SNOMED code for substance use or a substance use disorder within a year prior to the date of their inclusion diagnosis. The codes we used to identify substance use are given in eTable 5 in Supplement 1. We chose the broader term *PWUD* over *people who inject drugs*, as prior work has shown that *ICD* codes misclassify injecting drug use.^35^

### Confounders

We included individual-level and facility-level variables documented prior to discharge as potential confounders. At the individual level, we included age at diagnosis, sex, race and ethnicity, insurance coverage, individual Elixhauser comorbidities, length of hospital admission, and type of discharge. Race was identified as US census categories: American Indian or Alaska Native, Asian, Black or African American, Native Hawaiian or Other Pacific Islander, White, or multiracial group. We grouped individuals who were not identified in the prior categories as other race. Due to small numbers, we also grouped those of American Indian or Alaska Native, Asian, Native Hawaiian or Other Pacific Islander, or mixed racial group as other race. We classified ethnicity as Hispanic or Latino or non-Hispanic or non-Latino. Ethnicity and race were used as documented in the database and were included to identify potential disparities in access to laLGPs. We only included Elixhauser comorbidities codes from before the index hospitalization. For PWUD participants, we additionally included covariates for any history of cocaine, opioid, or methamphetamine use or disorder, history of injection drug use, and history of receiving a medication for opioid use disorder (methadone or buprenorphine). At the facility level, we included facility location by the first digit of US zip code, which divides the US into 10 geographic areas, and size in 500-bed increments. Covariates with missing values were coded as missing and this category was used in analyses.

### Outcomes

The primary outcome was a composite variable of clinical failure that included readmission, emergency department visit, or inpatient mortality or discharge to hospice within 90 days from the discharge date of the index encounter. All outcomes had to occur after the date of discharge of the initial index admission. A composite outcome was chosen to capture a broad range of clinically relevant events that may be indicative of treatment failure, consistent with prior infectious diseases research.^36,37,38^ We defined loss to follow-up as right-censored within 90 days of discharge from the index hospitalization, meaning the participant had no recorded clinical encounters (eg, outpatient visits, hospitalizations, or emergency department visits) after their last clinical encounter, and they did not meet any predefined end points within the 90-day postdischarge period.

### Statistical Analysis

We tabulated baseline covariates for those in the laLGP and SOC groups and used Fisher exact tests and independent samples *t* tests to compare the groups within the PWUD and non-PWUD strata. To estimate the association of initiating laLGP therapy within 10 days of hospital discharge with 90-day post-discharge clinical outcomes within the target emulation trial framework,^39^ we applied the clone-censor-weighting (CCW) approach.^40,41^ The CCW approach mitigates immortal time bias by cloning individuals at baseline and censoring clones upon treatment deviation, and it minimizes selection bias by applying inverse probability weights to adjust for differences between censored and uncensored patients. In the CCW approach, each individual was cloned at the time of discharge into 2 treatment strategies: one in which laLGP therapy was initiated within the 10-day grace period (laLGP group) and one in which SOC antibiotics were continued (SOC group). We censored laLGP clones on the day they did not receive laLGP therapy within the grace period or if they received SOC antibiotics during that time, whereas those in the SOC clone were censored on the day they received laLGP therapy. We estimated inverse probability of censoring weights at each time point using logistic regression models conditional on baseline covariates, including age, sex, race and ethnicity, insurance status, Elixhauser comorbidities, and facility characteristics. We truncated weights at the 99th percentile to limit extreme values and improve model stability. We then fitted weighted Cox proportional hazards models to estimate the association with the composite outcome within 90 days of discharge. We reported hazard ratios (HRs) and the difference in restricted mean survival time at 90 days. We derived 95% CIs for both indicators by performing 500 bootstrapped iterations, using the 2.5th and 97.5th percentiles of the bootstrap distribution as the lower and upper bounds, respectively. As sensitivity analyses, we fitted similar models with subsets by the qualifying diagnosis and comparing laLGP with vancomycin or cefazolin. We performed all analyses in R, version 4.0.2, within a Jupyter Notebook (R Program for Statistical Computing).^42^

## Results

### Cohort Description

**Figure 1.**
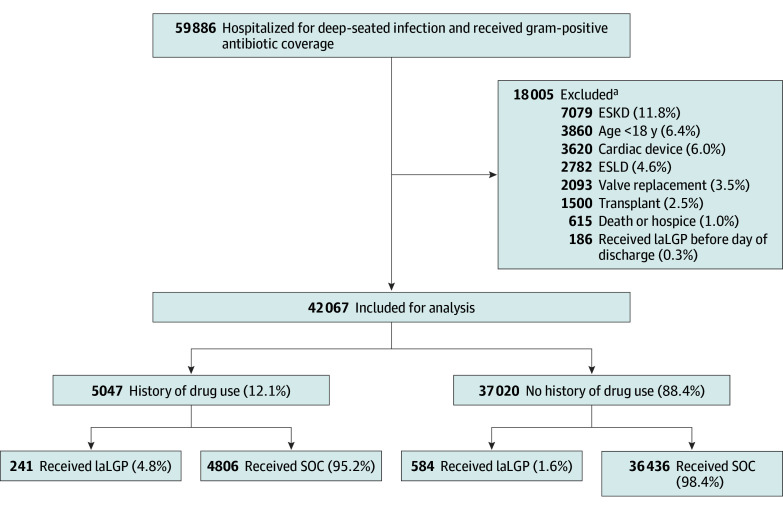
Study Flow Diagram ESKD, end-stage kidney disease; ESLD indicates end-stage liver disease; laLGP, long-acting lipoglycopeptide; and SOC, standard of care. ^a^Reasons are not mutually exclusive.

We identified a total of 59 886 individuals who met inclusion criteria. Reasons for exclusion (17 819 individuals [29.8%]) are detailed in Figure 1, with the most common reason being a history of end-stage kidney disease (ESKD) (7079 [11.8%]). Of 42 067 individuals included in our analysis, 5047 (12.1%) were included in the PWUD participants. Median age was 61 (IQR, 47-73) years; 17 343 participants (41.2%) were female and 24 704 (58.7%) were male. In terms of race, 4201 participants (10.5%) were Black, 32 905 (78.2%) were White, 1864 (4.4%) belonged to another racial group; and 3097 (7.4%) had unknown race. In terms of ethnicity, 6208 participants (14.8%) were Hispanic or Latino, with 34 668 (82.4%) non-Hispanic or non-Latino and 1191 (2.8%) unknown.

### Trends in laLGP Use

**Figure 2.**
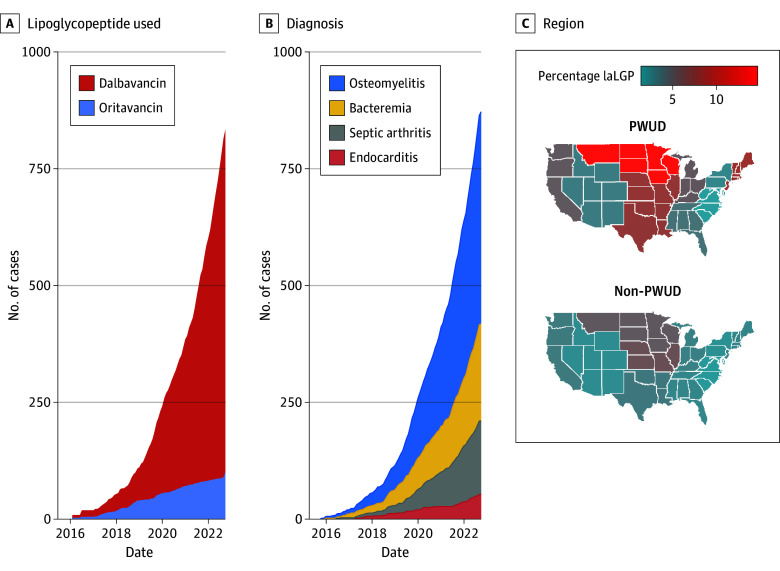
Temporal Patterns and Geographic Distribution of Long-Acting Lipoglycopeptide (laLGP) Use for Serious Bacterial Infections Data are from the US, October 1, 2015, to October 1, 2022. A, Patterns in laLGP use by specific drug. B, Patterns in laLGP use over time by infectious disease diagnosis. C, Percentage of serious bacterial infections treated with laLGP in different geographic regions. PWUD indicates people who use drugs.

In the entire sample, laLGPs were prescribed in 825 cases (2.0%), including 241 PWUD (4.8%) and 584 non-PWUD (1.6%) participants. Dalbavancin was the most common laLGP prescribed (733 [88.8%] of individuals who received laLGP). laLGPs were used most frequently for treatment of osteomyelitis (454 [55.0%]), followed by BSI (210 [24.5%]), septic arthritis (157 [19.0%]), and endocarditis (59 [7.2%]). Some individuals had multiple inclusion diagnoses. Figure 2 shows patterns over time by laLGP received and inclusion diagnosis and geographic use of laLGPs in the US.

### Sample Descriptions

Summary characteristics for the full sample of PWUD and non-PWUD participants are given in Table 1. For the PWUD group, the mean (SD) age was 45 (14) years, and most participants were male (2886 [57.2%]), White (4098 [81.2%]), and non-Hispanic or non-Latino (4345 [86.1%]). In the non-PWUD group, the mean (SD) age was 61 (17) years, and most participants were male (21 818 [58.9%]), White (28 807 [77.8%]), and non-Hispanic or non-Latino (30 323 [81.9%]). Missing data in the PWUD group were present for gender (2 [0.04%]), race (295 [5.8%]), ethnicity (129 [2.6%]), insurance (1045 [20.7%]), 1-digit zip code (3 [0.1%]), and hospital bed capacity (3 [0.1%]). In the non-PWUD group, missing data were present for gender (18 [0.05%]), race (2802 [7.6%]), ethnicity (1062 [2.9%]), insurance (7663 [20.7%]), zip code (31 [0.1%]), and hospital bed capacity (61 [0.2%]). In the PWUD group, there were no significant differences in the length of stay by treatment (12 [11] days for laLGP vs 13 [13] days for SOC; *P* = .80). The PWUD participants in the laLGP group were more frequently discharged home (169 [70.1%] vs 2223 [46.3%]) and less frequently to nursing facilities or rehabilitation (7 [2.9%] vs 1000 [20.8%]; *P* < .001) than the PWUD participants in the SOC group. Among non-PWUD participants, those in the laLGP group had similar lengths of stay (8 [7] vs 10 [14] days; *P* = .21) and a higher likelihood of discharge home (475 [81.3%] vs 21 720 [59.6%]; *P* < .001) and less likelihood of being discharged to a nursing home (36 [6.2%] vs 8333 [22.9%]; *P* < .001) than non-PWUD participants in the SOC group. There were no significant differences in loss to follow-up in the laLGP group compared with the SOC group among both PWUD (35 [14.5%] vs 709 [14.8%]; *P* > .99) and non-PWUD (124 [21.2%] vs 7013 [19.2%]; *P* = .20) participants. Among 5070 PWUD participants, the 3 most common terminal antibiotics in the SOC group were vancomycin (933 [18.4%]), cefazolin (848 [16.7%]), and cephalexin (554 [10.9%]). Among 36 436 non-PWUD participants, the 3 most common terminal antibiotics in the SOC group were cefazolin (7150 [19.6%]), vancomycin (6702 [18.4%]), and trimethoprim-sulfamethoxazole (572 [11.9%]). eTables 6 and 7 in Supplement 1 present a full list.

**Table 1.** Baseline Characteristics of Patients Admitted Due to a Serious Bacterial Infection Stratified by Drug Use Status and Prescription of laLGPs

Characteristic	PWUD status			Non-PWUD status		
	Treatment, No. (%)		*P* value^a^	Treatment, No. (%)		*P* value^a^
	SOC (n = 4806)	laLGP (n = 241)		SOC (n = 36 436)	laLGP (n = 584)	
Age, mean (SD), y	46 (15)	43 (13)	.02	62 (17)	54 (17)	<.001
Gender						
Male	2745 (57.1)	141 (58.5)	.70	21 419 (58.8)	399 (68.3)	<.001
Female	2059 (42.8)	100 (41.5)		14 999 (41.2)	185 (31.7)	
Unknown	2 (0.04)	0		18 (0.05)	0	
Race						
Black or African American	435 (9.1)	14 (5.8)	.13	3707 (10.2)	45 (7.7)	.06
White	3901 (81.2)	197 (81.7)		28 336 (77.8)	471 (80.7)	
Other^b^	190 (4.0)	15 (6.2)		1641 (4.5)	18 (3.1)	
Unknown	280 (5.8)	15 (6.2)		2752 (7.6)	50 (8.6)	
Ethnicity						
Hispanic or Latino	543 (11.3)	30 (12.4)	.80	5553 (15.2)	82 (14.0)	.70
Not Hispanic or Latino	4141 (86.2)	204 (84.6)		29 839 (81.9)	484 (82.9)	
Unknown	122 (2.5)	7 (2.9)		1044 (2.9)	18 (3.1)	
Insurance						
Medicaid	2102 (43.7)	104 (43.2)	.10	6092 (16.7)	144 (24.7)	<.001
Medicare	817 (17.0)	28 (11.6)		14 343 (39.4)	160 (27.4)	
Commercial insurance	325 (6.8)	15 (6.2)		5841 (16.0)	88 (15.1)	
Other	573 (11.9)	38 (15.8)		2598 (7.1)	91 (15.6)	
Unknown	989 (20.6)	56 (23.2)		7562 (20.8)	101 (17.3)	
Comorbidities						
Alcohol abuse	478 (9.9)	33 (13.7)	.06	1298 (3.6)	21 (3.6)	>.99
Blood loss anemia	60 (1.2)	1 (0.4)	.40	362 (1.0)	9 (1.5)	.20
Chronic peptic ulcer disease	51 (1.1)	4 (1.7)	.30	336 (0.9)	5 (0.9)	.90
Chronic pulmonary disease	851 (17.7)	43 (17.8)	>.9	5226 (14.3)	88 (15.1)	.60
Coagulation deficiency	233 (4.8)	11 (4.6)	.80	1748 (4.8)	27 (4.6)	.80
Congestive heart failure	363 (7.6)	17 (7.1)	.80	3780 (10.4)	48 (8.2)	.09
Deficiency anemias	984 (20.5)	62 (25.7)	.05	7068 (19.4)	119 (20.4)	.60
Depression	951 (19.8)	65 (27.0)	.007	3390 (9.3)	86 (14.7)	<.001
Diabetes with chronic complications	589 (12.3)	42 (17.4)	.02	6671 (18.3)	160 (27.4)	<.001
Diabetes without chronic complications	864 (18.0)	47 (19.5)	.50	10 649 (29.2)	203 (34.8)	.004
Fluid and electrolyte disorders	1507 (31.4)	82 (34.0)	.40	10 304 (28.3)	164 (28.1)	>.99
HIV and AIDS	105 (2.2)	7 (2.9)	.50	230 (0.6)	4 (0.7)	.80
Hypertension, complicated	94 (2.0)	5 (2.1)	.80	600 (1.6)	9 (1.5)	.80
Hypertension, uncomplicated	1325 (27.6)	73 (30.3)	.40	15 014 (41.2)	241 (41.3)	>.99
Hypothyroidism	219 (4.6)	11 (4.6)	>.99	3079 (8.5)	39 (6.7)	.13
Liver disease	390 (8.1)	34 (14.1)	.001	954 (2.6)	16 (2.7)	.90
Lymphoma	27 (0.6)	4 (1.7)	.06	486 (1.3)	11 (1.9)	.30
Metastatic cancer	51 (1.1)	1 (0.4)	.50	772 (2.1)	9 (1.5)	.30
Obesity	515 (10.7)	30 (12.4)	.40	4871 (13.4)	118 (20.2)	<.001
Other neurological disorders	765 (15.9)	47 (19.5)	.14	5084 (14.0)	66 (11.3)	.07
Paralysis	146 (3.0)	6 (2.5)	.60	1132 (3.1)	25 (4.3)	.11
Peripheral vascular disease	361 (7.5)	24 (10.0)	.20	3446 (9.5)	68 (11.6)	.07
Psychoses	734 (15.3)	50 (20.7)	.02	1521 (4.2)	36 (6.2)	.02
Pulmonary circulation disorders	322 (6.7)	20 (8.3)	.30	1137 (3.1)	22 (3.8)	.40
Kidney failure	225 (4.7)	15 (6.2)	.30	3182 (8.7)	43 (7.4)	.20
Rheumatoid arthritis or collagen vascular diseases	197 (4.1)	13 (5.4)	.30	1352 (3.7)	14 (2.4)	.09
Solid tumor without metastasis	170 (3.5)	4 (1.7)	.12	2959 (8.1)	35 (6.0)	.06
Valvular disease	337 (7.0)	26 (10.8)	.03	1724 (4.7)	28 (4.8)	>.9
Weight loss	381 (7.9)	22 (9.1)	.50	2162 (5.9)	43 (7.4)	.15
Substance use and/or disorder						
History of opioid use or disorder	3431 (71.4)	177 (73.4)	.50	NA	NA	NA
History of cocaine use or disorder	492 (10.2)	30 (12.4)	.30	NA	NA	NA
History of methamphetamine use or disorder	1254 (26.1)	87 (36.1)	<.001	NA	NA	NA
Injection drug use	857 (17.8)	29 (12.0)	.02	NA	NA	NA
MOUD						
No MOUD	2821 (58.7)	146 (60.6)	.80	NA	NA	NA
Methadone	1129 (23.5)	56 (23.2)		NA	NA	
Buprenorphine	856 (17.8)	39 (16.2)		NA	NA	
Infectious diseases diagnosis						
Blood stream infection, isolated	1844 (38.4)	59 (24.5)	<.001	17 782 (48.8)	151 (25.9)	<.001
Osteomyelitis	1533 (31.9)	111 (46.1)	<.001	12 382 (34.0)	343 (58.7)	<.001
Septic arthritis	774 (16.1)	54 (22.4)	.010	5113 (14.0)	103 (17.6)	.01
Endocarditis	907 (18.9)	34 (14.1)	.06	2087 (5.7)	19 (3.3)	.01
Hospital capacity, No. of beds						
<500	746 (15.5)	44 (18.3)	<.001	6372 (17.5)	109 (18.7)	<.001
500-999	1659 (34.5)	115 (47.7)		12 595 (34.6)	315 (53.9)	
≥1000	2398 (49.9)	82 (34.0)		17 408 (47.8)	160 (27.4)	
Unknown	3 (0.1)	0		61 (0.2)	0	
1-digit Zip code						
0: Connecticut, Massachusetts, Maine, New Hampshire, New Jersey, Puerto Rico, Rhode Island	543 (11.3)	51 (21.2)	<.001	3248 (8.9)	41 (7.0)	<.001
1: Delaware, New York, Pennsylvania	621 (12.9)	15 (6.2)		4162 (11.4)	27 (4.6)	
2: Maryland, North Carolina, South Carolina, Virginia, Washington, DC, West Virginia	514 (10.7)	7 (2.9)		2951 (8.1)	5 (0.9)	
3: Alabama, Florida, Georgia, Mississippi, Tennessee	520 (10.8)	19 (7.9)		4643 (12.7)	53 (9.1)	
4: Indiana, Kentucky, Michigan, Ohio	291 (6.1)	16 (6.6)		2409 (6.6)	40 (6.8)	
5: Iowa, Minnesota, Montana, North Dakota, South Dakota, Wisconsin	105 (2.2)	18 (7.5)		1300 (3.6)	58 (9.9)	
6: Illinois, Kansas, Missouri, Nebraska	378 (7.9)	33 (13.7)		3080 (8.5)	159 (27.2)	
7: Arkansas, Louisiana, Oklahoma, Texas	197 (4.1)	17 (7.1)		2385 (6.5)	49 (8.4)	
8: Arizona, Colorado, Idaho, New Mexico, Nevada, Utah, Wyoming	984 (20.5)	30 (12.4)		7658 (21.0)	60 (10.3)	
9: Alaska, California, Hawaii, Oregon, Washington	650 (13.5)	35 (14.5)		4569 (12.5)	92 (15.8)	
Unknown	3 (0.1)	0		31 (0.1)	0	

Abbreviations: laLGP, long-acting lipoglycopeptide; MOUD, medication for opioid use disorder; NA, not applicable; PWUD, person who uses drugs; SOC, standard of care antibiotics.

^a^ Calculated using Fisher exact test or 2-sided *t* tests.

^b^ Includes American Indian or Alaska Native, Asian, Native Hawaiian or Other Pacific Islander, and multiracial.

### Unadjusted Outcomes

Unadjusted clinical outcomes for the full sample are given in Table 2. Among PWUD participants, 2599 (51.5%) met the composite outcome of inpatient death, discharge to hospice, readmission, or emergency department visit within 90 days after discharge. PWUD participants in the laLGP group were less likely to meet the composite outcome within 90 days (107 [44.4%] vs 2492 [51.9%]; *P* = .02) and were less likely to be readmitted at 90 days (62 [25.7%] vs 1892 [39.4%]; *P* < .001) than PWUD participants in the SOC group. Among non-PWUD participants, 15 297 (41.3%) met the composite outcome within 90 days. Non-PWUD participants in the laLGP group were less likely to meet the composite outcome (185 [31.7%] vs 15 112 [41.5%]; *P* < .001) and were less likely to be readmitted (116 [19.9%] vs 11 360 [31.2%]; *P* < .001) than non-PWUD participants in the SOC group.

**Table 2.** Unadjusted 90-Day Outcomes by laLGP Use for Admissions Due to a Serious Bacterial Infection

Outcome	PWUD status				Non-PWUD status			
	Treatment, No. (%)			*P* value^a^	Treatment, No. (%)			*P* value^a^
	Overall (n = 5047)	SOC (n = 4806)	laLGP (n = 241)		Overall (n = 37 020)	SOC (n = 36 436)	laLGP (n = 584)	
Readmission, 90 d	1954 (38.7)	1892 (39.4)	62 (25.7)	<.001	11 476 (31.0)	11 360 (31.2	116 (19.9)	<.001
ED visit, 90 d	1352 (26.8)	1287 (26.8)	65 (27.0)	>.99	6837 (18.5)	6740 (18.5)	97 (16.6)	.20
Death or hospice, 90 d	5 (0.1)	5 (0.1)	0	>.99	39 (0.1)	38 (0.1)	1 (0.2)	.50
Composite of readmission, ED visit, or death or hospice, 90 d	2599 (51.5)	2492 (51.9)	107 (44.4)	.02	15 297 (41.3)	15 112 (41.5)	185 (31.7)	<.001

Abbreviations: ED, emergency department; laLGP, long-acting lipoglycopeptide; PWUD, person who uses drugs; SOC, standard of care antibiotics.

^a^ Calculated using Fisher exact test.

### Clone Censor Weighting Analysis

Table 3 shows results of the bootstrapped Cox proportional hazards models. Among both PWUD (HR, 1.01; 95% CI, 0.88-1.13) and non-PWUD (HR, 0.93; 95% CI, 0.86-1.00) participants, there were no differences between the laLGP and the SOC groups in the composite outcome. In the non-PWUD participants and individuals diagnosed with osteomyelitis, laLGP prescription had a protective association with the composite outcome compared with SOC (HR, 0.85; 95% CI, 0.76-0.96). In analyses comparing specific antibiotics, non-PWUD participants in the laLGP group had a lower risk of the composite outcome compared with prescription of vancomycin (HR, 0.84; 95% CI, 0.76-0.92) and cefazolin (HR, 0.87; 95% CI, 0.79-0.96). There were no significant differences in subanalyses among PWUD participants.

**Table 3.** Hazard Ratios and RMST for 90-Day Outcomes

Outcome	HR (95% CI)^a^	RMST, difference (95% CI), %^b^
**PWUD status**		
Entire cohort	1.01 (0.88 to 1.13)	−0.73 (−4.17 to 2.68)
BSI, isolated	1.06 (0.83 to 1.32)	−2.04 (−9.01 to 5.12)
Osteomyelitis	1.07 (0.88 to 1.25)	−2.82 (−8.12 to 2.57)
Septic arthritis	0.88 (0.71 to 1.07)	4.22 (−1.81 to 9.96)
Endocarditis	1.09 (0.84 to 1.35)	−3.67 (−12.59 to 5.28)
laLGP vs vancomycin	0.90 (0.77 to 1.03)	2.24 (−1.56 to 6.14)
laLGP vs cefazolin	0.97 (0.81 to 1.13)	0.66 (−3.05 to 4.66)
**Non-PWUD status**		
Entire cohort	0.93 (0.86 to 1.00)	1.76 (0.03 to 3.91)
BSI, isolated	0.99 (0.98 to 1.13)	0.55 (−3.36 to 4.23)
Osteomyelitis	0.85 (0.76 to 0.96)	3.28 (0.71 to 5.57)
Septic arthritis	0.93 (0.76 to 1.10)	−0.12 (−3.26 to 5.35)
Endocarditis	1.01 (0.96 to 1.13)	−4.03 (−20.86 to 10.6)
laLGP vs vancomycin	0.84 (0.76 to 0.92)	3.98 (1.78 to 6.00)
laLGP vs cefazolin	0.87 (0.79 to 0.96)	3.02 (0.76 to 5.14)

Abbreviations: BSI, bloodstream infection; HR, hazard ratio; laLGP, long-acting lipoglycopeptide; RMST, restricted mean survival time; PWUD, person who uses drugs.

^a^ Hazard ratios of less than 1 favor laLGPs and hazard ratios of more than 1 favor the comparator group (grouped standard of care antibiotics or vancomycin or cefazolin, where noted).

^b^ RMST difference of more than 0 favor laLGPs and RMST difference of less than 0 favor the comparator.

## Discussion

In this comparative effectiveness study, we examined utilization patterns and clinical effectiveness within a target trial emulation framework of laLGPs for the management of serious bacterial infections among PWUD and non-PWUD participants in a US national database. We found individuals who received laLGPs had similar 90-day treatment outcomes compared with those who received SOC antibiotics and across individual serious bacterial infections. This is an encouraging finding for the off-label use of laLGPs and adds to the evidence base supporting the effectiveness of laLGPs among PWUD and non-PWUD individuals.^17,18,19,20,21,22,23,24^

Our study suggests clinicians are adopting laLGPs to treat serious bacterial infections, although overall use remains limited. We were not able to discern reasons for choosing an laLGP over other antimicrobials. Of note, in our study, patients with a history of mental health disorders, namely depression or psychosis, were more likely to receive laLGP than SOC. Clinicians may infer that individuals with mental health issues may be less likely to adhere to daily antibiotic therapy and an laLGP may be ideal in this clinical situation. We also noted a higher percentage of serious bacterial infections were treated with laLGPs in the Midwestern and Western US compared with the Pacific West and the Northeast. This may suggest a preference for laLGPs in rural areas. OPAT requires frequent laboratory monitoring and close nurse, pharmacist, and physician follow-up, which may be more challenging in regions with limited access. As knowledge about oral antibiotics grows, research is needed to understand clinician and patient preferences for managing serious bacterial infections and to educate prescribers on laLGP benefits and adverse effects.^43,44,45^

Dalbavancin was the most frequently prescribed agent (88.8% of those receiving laLGPs), especially for treatment of osteomyelitis and BSI. This has important implications for our clinical effectiveness findings, as the results may not extrapolate to oritavancin. Our study was unable to evaluate why there was a preference for dalbavancin over oritavancin. One notable factor early in the introduction of laLGPs was the shorter infusion time of dalbavancin compared with oritavancin (30 minutes vs 3 hours). In March 2021, oritavancin was approved as a shorter infusion volume and time (1 hour).^46^ Further studies are necessary to assess whether this new formulation will increase use of oritavancin.

### Limitations

Several limitations of this study should be considered. Our large retrospective multicenter dataset may have inherent biases like misclassification of diagnoses or incomplete records. Reliance on *ICD* codes for case identification could introduce coding errors or variability across health care facilities. Our classification of PWUD based solely on diagnostic codes might misrepresent the true population, as clinicians may not necessarily diagnose substance use disorders or enter diagnostic codes into their electronic health record. There is a risk that a subset of patients classified as non-PWUD were PWUD. The lack of culture data required proxies for gram-positive infections, which may not accurately reflect the true microbiological profile. Despite adjusting for various covariates, residual confounding due to unmeasured or inaccurately measured factors remained possible. Antibiotic therapy adherence and completion of SOC therapy were not directly measurable in this retrospective analysis. This limitation is particularly relevant for PWUD individuals and those with other mental illness, among whom care pathways may vary substantially, especially when directing their own discharge.

## Conclusions

In this comparative effectiveness study of laLGPs, we observed that laLGPs may be effective as step-down options in treating serious gram-positive bacterial infections, offering comparable outcomes to SOC antibiotics in both PWUD and non-PWUD populations. Our study highlighted utilization patterns and supported the clinical effectiveness of laLGPs in serious bacterial infections among a diverse patient population. While awaiting a randomized clinical trial,^47^ large national databases could help clinicians understand laLGPs’ efficacy, especially for off-label use among PWUD individuals. Despite limitations, our findings suggested dalbavancin was an effective therapeutic option. Future research should compare patient and clinician preferences among intravenous treatments, laLGPs, and oral antibiotics, as well as the cost-effectiveness of each option. Clinically, laLGPs can be an effective alternative to standard antibiotic courses for serious bacterial infections.

## Notes

Supplement 1. **eTable 1.** Specification and Emulation of a Target Trial of Long-Acting Glycopeptides vs Standard of Care in Patients With Serious Bacterial Infections **eTable 2.** Inclusion Criteria Diagnostic Codes **eTable 3.** Exclusion Diagnostic Codes **eTable 4.** Exclusion Procedure Codes **eTable 5.** Codes to Identify People Who Use Drugs **eTable 6.** Antibiotic Characteristics for PWUD Group **eTable 7.** Antibiotic Characteristics for Non-PWUD Group

Supplement 2.
Data Sharing Statement

## References

[1] Fleischauer AT, Ruhl L, Rhea S, Barnes E. Hospitalizations for endocarditis and associated health care costs among persons with diagnosed drug dependence—North Carolina, 2010-2015. MMWR Morb Mortal Wkly Rep. 2017;66(22):569-573. doi:10.15585/mmwr.mm6622a1 28594786 PMC5720243

[2] Ronan MV, Herzig SJ. Hospitalizations related to opioid abuse/dependence and associated serious infections increased sharply, 2002-12. Health Aff (Millwood). 2016;35(5):832-837. doi:10.1377/hlthaff.2015.1424 27140989 PMC5240777

[3] Kievlan DR, Gukasyan M, Gesch J, Rodriguez RM. Clinical profile of injection drug users presenting to the ED. Am J Emerg Med. 2015;33(5):674-676. doi:10.1016/j.ajem.2015.02.020 25744147

[4] Degenhardt L, Peacock A, Colledge S, . Global prevalence of injecting drug use and sociodemographic characteristics and prevalence of HIV, HBV, and HCV in people who inject drugs: a multistage systematic review. Lancet Glob Health. 2017;5(12):e1192-e1207. doi:10.1016/S2214-109X(17)30375-3 29074409 PMC5683738

[5] Heger AH, Baden R, Spellberg B. When oral therapy can replace intravenous antibiotics-changing practice as new data emerge. JAMA Intern Med. 2023;183(6):505-506. doi:10.1001/jamainternmed.2023.0923 37067821

[6] Williams DN, Baker CA, Kind AC, Sannes MR. The history and evolution of outpatient parenteral antibiotic therapy (OPAT). Int J Antimicrob Agents. 2015;46(3):307-312. doi:10.1016/j.ijantimicag.2015.07.001 26233483

[7] Buehrle DJ, Shields RK, Shah N, Shoff C, Sheridan K. Risk factors associated with outpatient parenteral antibiotic therapy program failure among intravenous drug users. Open Forum Infect Dis. 2017;4(3):ofx102. doi:10.1093/ofid/ofx102 28680904 PMC5493937

[8] Ho J, Archuleta S, Sulaiman Z, Fisher D. Safe and successful treatment of intravenous drug users with a peripherally inserted central catheter in an outpatient parenteral antibiotic treatment service. J Antimicrob Chemother. 2010;65(12):2641-2644. doi:10.1093/jac/dkq355 20864497

[9] Dobson PM, Loewenthal MR, Schneider K, Lai K. Comparing injecting drug users with others receiving outpatient parenteral antibiotic therapy. Open Forum Infect Dis. 2017;4(4):ofx183. doi:10.1093/ofid/ofx183 29026870 PMC5632303

[10] Fanucchi L, Leedy N, Li J, Thornton AC. Perceptions and practices of physicians regarding outpatient parenteral antibiotic therapy in persons who inject drugs. J Hosp Med. 2016;11(8):581-582. doi:10.1002/jhm.2582 27043146

[11] Ti L, Ti L. Leaving the hospital against medical advice among people who use illicit drugs: a systematic review. Am J Public Health. 2015;105(12):e53-e59. doi:10.2105/AJPH.2015.302885 26469651 PMC4638247

[12] Garland A, Ramsey CD, Fransoo R, . Rates of readmission and death associated with leaving hospital against medical advice: a population-based study. CMAJ. 2013;185(14):1207-1214. doi:10.1503/cmaj.130029 23979869 PMC3787167

[13] Guskey MT, Tsuji BT. A comparative review of the lipoglycopeptides: oritavancin, dalbavancin, and telavancin. Pharmacotherapy. 2010;30(1):80-94. doi:10.1592/phco.30.1.80 20030476

[14] Saravolatz LD, Stein GE. Oritavancin: a long-half-life lipoglycopeptide. Clin Infect Dis. 2015;61(4):627-632. doi:10.1093/cid/civ311 25900171

[15] Billeter M, Zervos MJ, Chen AY, Dalovisio JR, Kurukularatne C. Dalbavancin: a novel once-weekly lipoglycopeptide antibiotic. Clin Infect Dis. 2008;46(4):577-583. doi:10.1086/526772 18199045

[16] Dunne MW, Puttagunta S, Sprenger CR, Rubino C, Van Wart S, Baldassarre J. Extended-duration dosing and distribution of dalbavancin into bone and articular tissue. Antimicrob Agents Chemother. 2015;59(4):1849-1855. doi:10.1128/AAC.04550-14 25561338 PMC4356775

[17] Thomas G, Henao-Martínez AF, Franco-Paredes C, Chastain DB. Treatment of osteoarticular, cardiovascular, intravascular-catheter–related and other complicated infections with dalbavancin and oritavancin: a systematic review. Int J Antimicrob Agents. 2020;56(3):106069. doi:10.1016/j.ijantimicag.2020.106069 32603683

[18] Cooper CC, Stein GE, Mitra S, Abubaker A, Havlichek DH. Long-acting lipoglycopeptides for the treatment of bone and joint infections. Surg Infect (Larchmt). 2021;22(8):771-779. doi:10.1089/sur.2020.413 33835882

[19] Morrisette T, Miller MA, Montague BT, Barber GR, McQueen RB, Krsak M. On- and off-label utilization of dalbavancin and oritavancin for Gram-positive infections. J Antimicrob Chemother. 2019;74(8):2405-2416. doi:10.1093/jac/dkz162 31322694

[20] Guleri A, More R, Sharma R, Wong M, Abdelrahman A. Use of dalbavancin in infective endocarditis: a case series. JAC Antimicrob Resist. 2021;3(3):dlab099. doi:10.1093/jacamr/dlab099 34396119 PMC8360293

[21] Bork JT, Heil EL, Berry S, . Dalbavancin use in vulnerable patients receiving outpatient parenteral antibiotic therapy for invasive gram-positive infections. Infect Dis Ther. 2019;8(2):171-184. doi:10.1007/s40121-019-0247-0 31054088 PMC6522607

[22] Ahiskali A, Rhodes H. Oritavancin for the treatment of complicated gram-positive infection in persons who inject drugs. BMC Pharmacol Toxicol. 2020;21(1):73. doi:10.1186/s40360-020-00452-z 33115540 PMC7594421

[23] Almangour TA, Perry GK, Terriff CM, Alhifany AA, Kaye KS. Dalbavancin for the management of gram-positive osteomyelitis: effectiveness and potential utility. Diagn Microbiol Infect Dis. 2019;93(3):213-218. doi:10.1016/j.diagmicrobio.2018.10.007 30396697

[24] Zambrano S, Paras ML, Suzuki J, . Real-world dalbavancin use for serious gram-positive infections: comparing outcomes between people who use and do not use drugs. Open Forum Infect Dis. 2024;11(4):ofae186. doi:10.1093/ofid/ofae186 38651139 PMC11034951

[25] Bassetti M, Labate L, Vena A, Giacobbe DR. Role or oritavancin and dalbavancin in acute bacterial skin and skin structure infections and other potential indications. Curr Opin Infect Dis. 2021;34(2):96-108. doi:10.1097/QCO.0000000000000714 33405480

[26] Ehwerhemuepha L, Carlson K, Moog R, . Cerner Real-World Data (CRWD)—a de-identified multicenter electronic health records database. Data Brief. 2022;42:108120. doi:10.1016/j.dib.2022.108120 35434225 PMC9006763

[27] Benchimol EI, Smeeth L, Guttmann A, ; RECORD Working Committee. The Reporting of Studies Conducted Using Observational Routinely-Collected Health Data (RECORD) statement. PLoS Med. 2015;12(10):e1001885. doi:10.1371/journal.pmed.1001885 26440803 PMC4595218

[28] Norris AH, Shrestha NK, Allison GM, . 2018 Infectious Diseases Society of America clinical practice guideline for the management of outpatient parenteral antimicrobial therapy. Clin Infect Dis. 2019;68(1):e1-e35. doi:10.1093/cid/ciy745 30423035

[29] AbbVie. Dalvance: coverage and reimbursement information. Published 2023. Accessed March 14, 2024. https://www.dalvance.com/coding-and-reimbursement?cid=ppc_CVe5398a3d1a984efd92609d98f073db5f&&msclkid=034147d5193213a3b9d4202159104a65&utm_source=bing&utm_medium=cpc&utm_campaign=Dalvance_HCP_Branded_Max_Brand_US-DAV-230028&utm_term=reimbursement%20for%20dalvance%20claim&utm_content=Brand%20-%20Exact&gclid=034147d5193213a3b9d4202159104a65&gclsrc=3p.ds

[30] Larance B, Dobbins T, Peacock A, . The effect of a potentially tamper-resistant oxycodone formulation on opioid use and harm: main findings of the National Opioid Medications Abuse Deterrence (NOMAD) study. Lancet Psychiatry. 2018;5(2):155-166. doi:10.1016/S2215-0366(18)30003-8 29336948

[31] Heslin KCEA, Steiner CA. Hospitalizations involving mental and substance use disorders among adults, 2012. Statistical Brief 191. In: *Healthcare Cost and Utilization Project (HCUP) Statistical Briefs*. Agency for Healthcare Research and Quality; 2015. Accessed January 6, 2024. https://www.ncbi.nlm.nih.gov/books/NBK310986/table/sb191.t4/ 26290939

[32] Lewer D, Padmanathan P, Qummer Ul Arfeen M, . Healthcare use by people who use illicit opioids (HUPIO): development of a cohort based on electronic primary care records in England. Wellcome Open Res. 2021;5:282. doi:10.12688/wellcomeopenres.16431.2 33659712 PMC7901498

[33] Fingar KROP. Opioid-related and stimulant-related adult inpatient stays, 2012–2018. In: *Healthcare Cost and Utilization Project (HCUP) Statistical Briefs*. Agency for Healthcare Research and Quality; 2021. Accessed January 6, 2024. https://www.ncbi.nlm.nih.gov/books/NBK568393/table/sb271.tab6/

[34] Centers for Diseases Control and Prevention. CDC All Drug Overdose (version 2). Accessed January 6, 2024. https://cdn.ymaws.com/www.cste.org/resource/resmgr/overdose_surveillance/CDC_All_Drug_Definition_v2.pdf

[35] Goodman-Meza D, Tang A, Aryanfar B, . Natural language processing and machine learning to identify people who inject drugs in electronic health records. Open Forum Infect Dis. 2022;9(9):ofac471. doi:10.1093/ofid/ofac471 36168546 PMC9511274

[36] Rebold N, Alosaimy S, Pearson JC, . Dalbavancin sequential therapy for gram-positive bloodstream infection: a multicenter observational study. Infect Dis Ther. 2024;13(3):565-579. doi:10.1007/s40121-024-00933-2 38427289 PMC10965835

[37] Antosz K, Al-Hasan MN, Lu ZK, . Clinical utility and cost effectiveness of long-acting lipoglycopeptides used in deep-seated infections among patients with social and economic barriers to care. Pharmacy (Basel). 2021;10(1):1. doi:10.3390/pharmacy10010001 35076601 PMC8788434

[38] Texidor WM, Miller MA, Molina KC, . Oritavancin as sequential therapy for gram-positive bloodstream infections. BMC Infect Dis. 2024;24(1):127. doi:10.1186/s12879-023-08725-8 38267844 PMC10807122

[39] Hernán MA, Robins JM. Using big data to emulate a target trial when a randomized trial is not available. Am J Epidemiol. 2016;183(8):758-764. doi:10.1093/aje/kwv254 26994063 PMC4832051

[40] Gaber CE, Hanson KA, Kim S, Lund JL, Lee TA, Murray EJ. The clone-censor-weight method in pharmacoepidemiologic research: foundations and methodological implementation. Curr Epidemiol Rep. 2024;11(3):164-174. doi:10.1007/s40471-024-00346-2

[41] Maringe C, Benitez Majano S, Exarchakou A, . Reflection on modern methods: trial emulation in the presence of immortal-time bias: assessing the benefit of major surgery for elderly lung cancer patients using observational data. Int J Epidemiol. 2020;49(5):1719-1729. doi:10.1093/ije/dyaa057 32386426 PMC7823243

[42] R Core Team. R: A language and environment for statistical computing. R Foundation for Statistical Computing. 2023. Accessed February 28, 2025. https://www.R-project.org/

[43] Pries-Heje MM, Wiingaard C, Ihlemann N, . Five-year outcomes of the Partial Oral Treatment of Endocarditis (POET) trial. N Engl J Med. 2022;386(6):601-602. doi:10.1056/NEJMc2114046 35139280

[44] Iversen K, Ihlemann N, Gill SU, . Partial oral versus intravenous antibiotic treatment of endocarditis. N Engl J Med. 2019;380(5):415-424. doi:10.1056/NEJMoa1808312 30152252

[45] Li HK, Rombach I, Zambellas R, ; OVIVA Trial Collaborators. Oral versus intravenous antibiotics for bone and joint infection. N Engl J Med. 2019;380(5):425-436. doi:10.1056/NEJMoa1710926 30699315 PMC6522347

[46] Hoover RK, Krsak M, Molina KC, Shah K, Redell M. Kimyrsa, an oritavancin-containing product: clinical study and review of properties. Open Forum Infect Dis. 2022;9(5):ofac090. doi:10.1093/ofid/ofac090 35392455 PMC8982769

[47] Turner NA, Zaharoff S, King H, ; Antibacterial Resistance Leadership Group (ARLG). Dalbavancin as an option for treatment of *S aureus bacteremia* (DOTS): study protocol for a phase 2b, multicenter, randomized, open-label clinical trial. Trials. 2022;23(1):407. doi:10.1186/s13063-022-06370-1 35578360 PMC9109297

