# Supplementary material for: Comparative Effectiveness of Long-Acting Lipoglycopeptides vs Standard-of-Care Antibiotics in Serious Bacterial Infections
Source: JAMA Netw Open. 2025 May 21;8(5):e2511641. doi: 10.1001/jamanetworkopen.2025.11641 (PMC12096263; doi:10.1001/jamanetworkopen.2025.11641)
Supplement: Supplement 1. — eTable 1. Specification and Emulation of a Target Trial of Long-Acting Glycopeptides vs Standard of Care in Patients With Serious Bacterial Infections eTable 2. Inclusion Criteria Diagnostic Codes eTable 3. Exclusion Diagnostic Codes eTable 4. Exclusion Procedure Codes eTable 5. Codes to Identify People Who Use Drugs eTable 6. Antibiotic Characteristics for PWUD Group eTable 7. Antibiotic Characteristics for Non-PWUD Group [file jamanetwopen-e2511641-s001.pdf]

Supplementary Online Content

Goodman-Meza D, Weiss RE, Poimboeuf ML, et al. Comparative effectiveness of long-acting lipoglycopeptides vs standard-of-care antibiotics in serious bacterial infections. *JAMA Netw Open.* 2025;8(5):e2511641. doi:10.1001/jamanetworkopen.2025.11641

eTable 1. Specification and Emulation of a Target Trial of Long-Acting Glycopeptides vs Standard of Care in Patients With Serious Bacterial Infections
eTable 2. Inclusion Criteria Diagnostic Codes
eTable 3. Exclusion Diagnostic Codes
eTable 4. Exclusion Procedure Codes
eTable 5. Codes to Identify People Who Use Drugs
eTable 6. Antibiotic Characteristics for PWUD Group
eTable 7. Antibiotic Characteristics for Non-PWUD Group

This supplementary material has been provided by the authors to give readers additional information about their work.

© 2025 Goodman-Meza D et al. *JAMA Network Open*.

eTable 1. Specification and Emulation of a Target Trial of Long-Acting Glycopeptides vs Standard of Care in Patients With Serious Bacterial Infections

Component	Target trial	Emulated trial using real-world data
**Design**	Multicentre open-label two-parallel arm superiority randomised trial.	
**Aim**	Estimate the effect of receiving long acting glycopeptides compared to standard of care in participants diagnosed with a serious bacterial infection on post-discharge outcomes	Same
**Eligibility**	1) 18 year or older;	Same
	2) Serious bacterial infections defined as a diagnosis of endocarditis, BSI, osteomyelitis, or septic arthritis;	Same
	3) Hospitalized	Diagnosis associated to a emergency room or hospitalization
	4) Gram-positive organism causing infection;	4) Received at least 7 days of gram-positive antibacterial coverage or long acting glycopeptide (dalbavancin or oritavancin) [1]
	5) Expected to survive until discharge	5) Survived until discharge
		6) Time of diagnosis between 1 October 2015 and 1 October 2022 [2]
**Exclusions**	1) had a concomitant central nervous system infection;	Same
	2) endocarditis requiring early surgical intervention (less than 10 days from initial diagnosis date);	Same
	3) presence of a prosthetic heart valve;	Same
	4) presence of a cardiac device (implantable cardiac defibrillator, pacemaker);	Same
	5) presence of a transplanted organ (kidney, heart, lung, liver, pancreas, bone marrow);	Same
	6) end-stage renal disease or dialysis;	Same
	7) end-stage liver disease;	Same
	8) polymicrobial gram positive/gram negative infection	8) received end of therapy antibiotics not typical for gram positive organisms (e.g., broad spectrum antibiotic that cover gram negative organisms)
	9) received laLGP prior to enrolment	9) received laLGP prior to discharge day
**Treatment strategies**	1. Long acting glycopeptide (dalbavancin or oritavancin)<br>2. Standard of care antibiotics	Same

© 2025 Goodman-Meza D et al. *JAMA Network Open*.

**Treatment assignment**	Patients are randomly assigned to either strategy once clinically stable.	Patients are non-randomly assigned to a treatment strategy. Randomisation is emulated via cloning of patients in both arms.
**Treatment implementation**	None	10 day grace period from discharge
**Primary outcome**	At 90 days post-discharge, composite outcome of:<br>1) Readmission,<br>2) Emergency room visit,<br>3) Death or hospice discharge	Same
**Type of outcome**	Composite; time to event	Same
**Follow up**	Follow up starts at discharge	Follow-up starts at discharge
**Censoring**	Loss to follow up, administrative censoring	Loss to follow up, administrative censoring, artificial censoring
**Stratums**	1) Persons who use drugs (PWUD)<br>2) Persons who do not use drugs	Same
**Adjustment variables (all at baseline prior to exposure)**		1) Age at diagnosis;<br>2) sex;<br>3) race;<br>4) ethnicity;<br>5) insurance coverage;<br>6) individual Elixhauser comorbidities;<br>7) facility location by 1-digit U.S. zip code,<br>8) facility bed size.<br><br>For PWUD stratum, additionally:<br>1) History of methamphetamine, opioid, or cocaine use or disorder<br>2) Medications for opioid use disorder
**Causal contrast**	*Intention to treat and Per protocol analysis*	*Per protocol*: we do not know what the intention to treat was from the data; In each arm of the emulated trial, patients who deviate from the protocol are censored at their time of deviation
**Estimands**	Differences in 90-day outcomes<br><br>90-day survival and restricted mean survival time at 90 days between arms	Same
**Notes:**		

© 2025 Goodman-Meza D et al. *JAMA Network Open*.

[1] Cerner Real World data did not contain culture data. Discharge antibiotics used in lieu to determine type of organism treated.
[2] Time period when long-acting glycopeptides were available in the United States.

© 2025 Goodman-Meza D et al. *JAMA Network Open*.

eTable 2. Inclusion Criteria Diagnostic Codes

Variable name	ICD-10		ICD-9	
	Code	Diagnosis	Code	Diagnosis
Endocarditis	I33.X<br>I38<br>I39	Acute and subacute infective endocarditis<br>Endocarditis, valve unspecified<br>Endocarditis and heart valve disorders in diseases classified elsewhere	421.0<br>421.1<br>421.9<br>424.90<br>424.91<br>424.99	Acute and subacute bacterial endocarditis<br>Acute and subacute infective endocarditis in diseases classified elsewhere<br>Acute endocarditis, unspecified<br>Endocarditis, valve unspecified, unspecified cause<br>Endocarditis in diseases classified elsewhere<br>Other endocarditis, valve unspecified
Bacteremia	R78.81	Bacteremia NOS	790.7	Bacteremia
Septic arthritis	M00.X	Pyogenic arthritis	711.0X<br>711.4X<br>711.9X	Pyogenic arthritis<br>Arthropathy associated with other bacterial diseases<br>Unspecified infective arthritis
Osteomyelitis	M86.X<br>M46.2<br>M46.21<br>M46.22<br>M46.3	Osteomyelitis<br>Osteomyelitis of vertebra<br>Osteomyelitis of vertebra, occipito-atlanto-axial region<br>Osteomyelitis of vertebra, cervical region<br>Infection of intervertebral disc (pyogenic)	730.0X<br>730.1X<br>730.2X<br>730.8X<br>730.9X	Acute osteomyelitis<br>Chronic osteomyelitis<br>Unspecified osteomyelitis<br>Other infections involving bone in diseases classified elsewhere<br>Unspecified infection of bone

© 2025 Goodman-Meza D et al. *JAMA Network Open*.

eTable 3. Exclusion Diagnostic Codes

Variable name	ICD-10		ICD-9		Time frame
	Code	Diagnosis	Code	Diagnosis	
Other types of endocarditis	A32.82<br>A39.52<br>A54.83<br>B33.21<br>B37.6<br>I01.1<br>M32.11<br>A52.06	Listerial<br>Meningococcal<br>Gonococcal heart infection<br>Viral<br>Candidal<br>Acute rheumatic<br>Endocarditis in systemic lupus erythematosus<br>Symptomatic cardiovascular syphilis	036.42<br>074.22<br>093.2x<br>098.84<br>112.81<br>115.04<br>115.14<br>115.94	Meningococcal<br>Coxsackie carditis (viral)<br>Syphilitic endocarditis<br>Gonococcal<br>Candidal<br>Infection by Histoplasma capsulatum<br>Infection by Histoplasma duboisii<br>Histoplasmosis, unspecified,	Concurrent (+/- 15 days from inclusion diagnosis)
Central nervous system infections	A39.51<br>G06.X<br>G00.X<br>G01<br>B37.5	Meningococcal encephalitis<br>Intracranial and intraspinal abscess and granuloma<br>Bacterial meningitis, not elsewhere classified<br>Meningitis in bacterial diseases classified elsewhere<br>Fungal Meningitis	320.X<br>324.X	Bacterial meningitis<br>Intracranial and intraspinal abscess	Concurrent (+/- 15 days from inclusion diagnosis)
End stage renal disease	N18.6<br>N18.5	End stage renal disease<br>Chronic kidney disease, stage 5	585.6<br>585.5	End stage renal disease<br>Chron kidney dis stage V	Prior (anytime before inclusion diagnosis)
End stage liver disease	K72.1X<br>K74.X<br>K70.2<br>K70.3	Chronic hepatic failure<br>Fibrosis and cirrhosis of liver<br>Alcoholic fibrosis and sclerosis of liver	572.8<br>571.5<br>571.2	Other sequelae of chronic liver disease<br>Cirrhosis of liver without mention of alcohol	Prior (anytime before inclusion diagnosis)

© 2025 Goodman-Meza D et al. *JAMA Network Open*.

Variable name	ICD-10		ICD-9		Time frame
	Code	Diagnosis	Code	Diagnosis	
	K70.4	Alcoholic cirrhosis of liver Alcoholic hepatic failure		Alcoholic cirrhosis of liver	
Prosthetic valves	Z95.2<br>Z95.3<br>Z95.4<br>T82.0<br>T82.6	Presence of a Prosthetic Heart Valve<br>Presence of xenogenic heart valve<br>Presence of other heart-valve replacemet<br>Mechanical complication of heart valve prosthesis<br>Infection and inflammatory reaction due to cardiac valve prosthesis	V43.3<br>V42.2<br>996.02<br>996.61	Heart valve replaced by other means<br>Heart valve replaced by transplant<br>Mechanical complication due to heart valve prosthesis<br>Infection and inflammatory reaction due to cardiac device, implant, and graft	Prior (anytime before inclusion diagnosis)
Other cardiac devices (ICD, pacemaker)	Z95.0<br>Z95.81<br>T82.1	Presence of cardiac pacemaker<br>Presence of other cardiac implants and grafts<br>Mechanical complication of cardiac electronic device	V45.0<br>996.01<br>996.04	Cardiac device in situ<br>Mechanical complication due to cardiac pacemaker (electrode)<br>Mechanical complication of automatic implantable cardiac defibrillator	Prior (anytime before inclusion diagnosis)
Transplant	Z94.0<br>Z94.1<br>Z94.2<br>Z94.3<br>Z94.4	Kidney transplant status<br>Heart transplant status<br>Lung transplant status<br>Heart and lungs transplant status	V42.0<br>V42.1<br>V42.2<br>V42.6	Kidney replaced by transplant<br>Heart replaced by transplant<br>Heart valve replaced by transplant<br>Lung replaced by transplant	Prior (anytime before inclusion diagnosis)

© 2025 Goodman-Meza D et al. *JAMA Network Open*.

Variable name	ICD-10		ICD-9		Time frame
	Code	Diagnosis	Code	Diagnosis	
	Z94.8<br>Z94.81<br>Z94.83<br>Z94.84<br>T86X<br>Z48.2	Liver transplant status<br>Other transplanted organ and tissue status<br>Bone marrow transplant status<br>Pancreas transplant status<br>Stem cells transplant status<br>Complications of transplanted organs and tissue<br>Encounter for aftercare following organ transplant	V42.7<br>V42.8<br>V42.81<br>V42.82<br>V42.83<br>996.8<br>V58.44	Liver replaced by transplant<br>Other specified organ or tissue replaced by transplant<br>Bone marrow replaced by transplant<br>Peripheral stem cells replaced by transplant<br>Pancreas replaced by transplant<br>Complications of transplanted organ<br>Aftercare following organ transplant	

© 2025 Goodman-Meza D et al. *JAMA Network Open*.

eTable 4. Exclusion Procedure Codes

Associated Procedure(s)	CPT Code(s)	ICD-10-PCS Code(s)	ICD-9 Procedure Code(s)	Time frame
End Stage Renal Disease				
Hemodialysis	36558<br>36800<br>36810<br>36815<br>Insertion of cannula for hemodialysis<br>90935<br>90937<br>Hemodialysis	5A1D<br>Urinary ultrafiltration<br><br>02H633Z<br>02HV33Z<br>0JH63XZ<br>Insertion of catheter	39.95<br>Hemodialysis	Prior (anytime before inclusion diagnosis)
Peritoneal dialysis	90945<br>90947<br>Dialysis other than hemodialysis<br><br>49324<br>49481<br>49421<br>Insertion peritoneal catheter	3E1M39Z<br><br><br>0WHG03Z<br>0WHG33Z<br>0WHG43Z<br>Insertion infusion device in peritoneal cavity	54.98 Peritoneal dialysis	Prior (anytime before inclusion diagnosis)
Arteriovenous fistula - graft creation	36818<br>36819<br>36820<br>36821<br>36825<br>36830<br>36832<br>36833<br>36838<br>Arteriovenous anastomosis	031T0ZZ<br>031V0DZ	39.27 Arteriovenostomy for renal dialysis<br>39.42 Revision of arteriovenous shunt for renal dialysis<br>39.43 Removal of arteriovenous shunt for renal dialysis	Prior (anytime before inclusion diagnosis)
Transplant				
Kidney	50300<br>50320<br>50323<br>50325	0TY00Z0 0TY00Z1<br>0TY00Z2 0TY10Z0<br>0TY10Z1 0TY10Z2<br>0TYK0Z0 0TYK0Z1	55.6 Kidney transplantation	Prior (anytime before

© 2025 Goodman-Meza D et al. *JAMA Network Open*.

Associated Procedure(s)	CPT Code(s)	ICD-10-PCS Code(s)	ICD-9 Procedure Code(s)	Time frame
	50327<br>50328<br>50329<br>50340<br>50360<br>50365<br>50370<br>50380<br>S2065	0TYL0Z0 0TYL0Z1 (combined kidney and pancreas)	55.61 Transplant from live related donor<br>55.69 Kidney transplant NEC	inclusion diagnosis)
Pancreas	48160<br>48550<br>48551<br>48552<br>48554<br>48556<br>S2065<br>S2102<br>0584T<br>0585T<br>0586T	0FYG0Z0<br>0FYG0Z1<br>0FYG0Z2	52.8 Transplant Of Pancreas	Prior (anytime before inclusion diagnosis)
Liver	47133<br>47135<br>47140<br>47141<br>47142<br>47143<br>47144<br>47145<br>47146<br>47147<br>47399<br>S2152	0FY00Z0<br>0FY00Z1<br>0FY00Z2	50.5 Liver Transplant	Prior (anytime before inclusion diagnosis)
Heart	33927<br>33928<br>33929<br>33930<br>33933<br>33935<br>33940<br>33944<br>33945<br>S2152	02YA0Z0<br>02YA0Z1<br>02YA0Z2<br>02RK0JZ<br>02RL0JZ	37.51 Heart Transplantation	Prior (anytime before inclusion diagnosis)
Lung	32850<br>32851	0BYC0Z0 0BYC0Z1 0BYC0Z2 0BYD0Z0	33.5 Lung Transplant	Prior (anytime

© 2025 Goodman-Meza D et al. *JAMA Network Open*.

Associated Procedure(s)	CPT Code(s)	ICD-10-PCS Code(s)	ICD-9 Procedure Code(s)	Time frame
	32852<br>32853<br>32854<br>32855<br>32856<br>S2060<br>S2061	0BYD0Z1 0BYD0Z2<br>0BYF0Z0 0BYF0Z1<br>0BYF0Z2 0BYG0Z0<br>0BYG0Z1 0BYG0Z2<br>0BYH0Z0 0BYH0Z1<br>0BYH0Z2 0BYJ0Z0<br>0BYJ0Z1 0BYJ0Z2<br>0BYK0Z0 0BYK0Z1<br>0BYK0Z2 0BYL0Z0<br>0BYL0Z1 0BYL0Z2<br>0BYM0Z0 0BYM0Z1<br>0BYM0Z2	33.6 Combined heart-lung transplantation	before inclusion diagnosis)
Intestine	44132<br>44133<br>44135<br>44136<br>44137	0DY60Z0 0DY60Z1<br>0DY80Z0 0DY80Z1<br>0DY80Z2 0DYE0Z0<br>0DYE0Z1 0DYE0Z2<br>0DYF0Z0 0DYF0Z1	46.97: Intestinal transplant<br>46.98: Combined liver-intestine transplant	Prior (anytime before inclusion diagnosis)
Bone marrow	38205-38215<br>38231-38231<br>38240-38243<br>86915-86915<br>G0267-G0267<br>S2140-S2140	XW033C3 XW043C3<br>30230AZ 30230G0<br>30230Y0 30233AZ<br>30233G0 30233Y0<br>30243AZ 30243G0<br>30243Y0 30253G0<br>30253Y0 30263G0<br>30263Y0 30240AZ<br>30240G0 30240Y0<br>30250G0 30250Y0<br>30260G0 30260Y0<br>30230G2 30230G3<br>30230G4 30230X0<br>30230X2 30230X3<br>30230X4 30230Y2<br>30230Y3 30230Y4<br>30233G2 30233G3<br>30233G4 30233X2<br>30233X3 30233X4<br>30233Y2 30233Y3<br>30233Y4 30240G2<br>30240G3 30240G4<br>30240X0 30240X2<br>30240X3 30240X4<br>30240Y2 30240Y3<br>30240Y4 30243G2	41.0 Bone Marrow or Hematopoietic Stem Cell Transplant	Prior (anytime before inclusion diagnosis)

Associated Procedure(s)	CPT Code(s)	ICD-10-PCS Code(s)	ICD-9 Procedure Code(s)	Time frame
		30243G3 30243G4 30243X2 30243X3 30243X4 30243Y2 30243Y3 30243Y4 30250G1 30250X0 30250X1 30250Y1 30260G1 30260X0 30260X1 30260Y1 30233X0 30243X0 30253G1 30253X0 30253X1 30253Y1 30263G1 30263X0 30263X1 30263Y1		
Cardiac related				
Pacemaker /Implantable cardiac defibrillator	33206 33207 33208 33202 33203 33215 33216 33217 33218 33220 33223 33224 33225 33230 33231 33240 33241 33243 33244 33249 33262 33263 33264 33270 33271 33272 33273 C7537 C7538 C7539	02HK0KZ 02HK3KZ 02HK4KZ 02HL0KZ 02HL3KZ 02HL4KZ 02HL0MZ 02HL3MZ 02HL4MZ 02H60KZ 02H63KZ 02H64KZ 02H70KZ 02H73KZ 02H74KZ 0JH60AZ 0JH63AZ 0JH80AZ 0JH83AZ 0JH606Z 0JH636Z 0JH607Z 0JH637Z 0JH608Z 0JH638Z 0JH60GZ 0JH63GZ 0JH60JZ 0JH63JZ 0JH60PZ 0JH63PZ 0JH60QZ 0JH63QZ 0JH60KZ 0JH63KZ 0JPT0ZZ 0JPT3ZZ 0JPT4ZZ 0JH60LZ 0JH63LZ 0JH60MZ 0JH63MZ 0JH60NZ 0JH63NZ	37.52 Implantation of total internal biventricular heart replacement system 37.8X Insertion, Replacement, Removal And Revision Of Pacemaker Device	Prior (anytime before inclusion diagnosis)

© 2025 Goodman-Meza D et al. *JAMA Network Open*.

Associated Procedure(s)	CPT Code(s)	ICD-10-PCS Code(s)	ICD-9 Procedure Code(s)	Time frame
	C7540<br>G0448			
Valve repair or replacement	33370<br>33390<br>33391<br>33405<br>33406<br>33410<br>33411<br>33412<br>33413<br>34440<br>Aortic valve related<br><br>33418<br>33419<br>33420<br>33422<br>33425<br>33426<br>33427<br>33430<br>Mitral related<br><br>33460<br>33463<br>33464<br>33465<br>33468<br>Tricuspid related<br><br>33471<br>33474<br>33475<br>33476<br>33477<br>33478<br>Pulmonary valve related	02RFx<br>02RGx 02RHx<br>02RJx<br>Replacement of valve<br><br>02QFx 02QGx<br>02QHx<br>02QJx<br>Repair valve, open approach	35.00<br>35.01<br>35.02<br>35.03<br>35.04<br>Closed heart valvuloplasty<br>35.1X Open heart valvuloplasty without replacement<br>35.2X Open and other replacement of heart valve	Prior (anytime before inclusion diagnosis) and within 10 days after inclusion diagnosis

© 2025 Goodman-Meza D et al. *JAMA Network Open*.

eTable 5. Codes to Identify People Who Use Drugs

Diagnosis category	ICD-9	ICD-10	SNOMED CT
Opioid related codes	965.00<br>965.01<br>965.02<br>965.09<br>970.1<br>304.0X<br>304.7X<br>305.5X<br>E850.0<br>E850.1<br>E850.2<br>E940.1	T40.0<br>T40.1<br>T40.2<br>T40.3<br>T40.4<br>F11X	297199006 11196001 74264003 216469009 295174006 295175007 295176008 242829007 13187008 216463005 290183003 290182008 47836003 60199004 68099003 216464004 216466002 216468001 241749009 242253008 242828004 242831003 290171000 290172007 290174008 290175009 290176005 290177001 290179003 290180000 290193005 290194004 290201006 290202004 290203009 290204003 295161000 295163002 295164008 295166005 295167001 295168006 295169003 295170002 295171003 295172005 295173000 295184007 295185008 295186009 295187000 295193008 295194002 295195001 295196000 295213004 297199006 231477003 75544000 191865004 191820008 426001001 231478008 231479000 231480002 77721001 5602001 191909007 191819002 145121000119106 191912005 191913000
Cocaine related codes	970.81<br>304.2X<br>305.6X<br>E938.5	T40.5<br>F14X	296321004 296324007 296322006 296323001 296325008 296326009 296327000 296328005 31956009 191831000 191832007 27956007 78267003 145101000119102 191916008 191918009 191919001 428493006
Methamphetamine related codes	969.70<br>969.71<br>969.72<br>969.73<br>969.79<br>304.4X<br>305.7X	T43.6<br>F15X	20260003 21647008 45775001 61803000 212670004 216558009 216559001 242257009 291241005 291242003 291258000 291259008 291260003 296290006 296291005 296292003 296293008 296294002 296317006 296318001 296329002 426873000 275471001 21647008 191843004 191844005 191877009 45421006 84758004 191924003 191925002 268648009 275471001 427205009 428659002 429692000 699449003

© 2025 Goodman-Meza D et al. *JAMA Network Open*.

Database	Terms/Codes	Descriptors
SNOMED	75544000	Opioid dependence
	699449003	Methamphetamine abuse
	228388006	Intravenous drug user
	5602001	Opioid abuse
	371435006	History of drug abuse
	26416006	Drug abuse
	426873000	Methamphetamine dependence
	191819002	Continuous opioid dependence
	361055000	Misuses drugs
	724653003	Opioid dependence with current use
	16464661000119100	History of intravenous drug abuse
	78267003	Cocaine abuse
	231477003	Heroin dependence
	70545002	Narcotic drug user
	191816009	Drug dependence
	11061003	Psychoactive substance use disorder
	191909007	Nondependent opioid abuse
	1081000119105	Opioid dependence, on agonist therapy
	441527004	Stimulant abuse
	55680006	Drug overdose
	6525002	Dependent drug abuse
	27660001000004100	History of methamphetamine abuse
	307052004	Illicit drug use
	31956009	Cocaine dependence
	49540005	Non dependent drug abuse
	733461000	Cocaine user
	1471000119103	Drug abuse in remission
	191821007	Opioid dependence in remission
	191865004	Combined opioid with other drug dependence
	228373001	Drug addict
	442406005	Stimulant dependence
	169941005	Maternal drug abuse
	426001001	Fentanyl dependence
	741063003	Illicit drug injection in last 12 months
	12398571000119100	Methamphetamine intoxication
	191833002	Cocaine dependence in remission
	191934007	Nondependent mixed drug abuse
	231481003	Controlled drug dependence
	295174006	Heroin overdose
	387341002	Heroin
	428493006	Crack cocaine misuse
	59274003	Intentional drug overdose
	59369008	Accidental drug overdose
	110281001	Chronic drug abuse
	1239828100011910 0	Methamphetamine withdrawal

© 2025 Goodman-Meza D et al. *JAMA Network Open*.

Database	Terms/Codes	Descriptors
	12398321000119100	Mood disorder caused by methamphetamine
	13187008	Poisoning by heroin
	14816004	Cocaine product
	153501000119105	Drug dependence, episodic
	191845006	Amphetamine or psychostimulant dependence in remission
	191914006	Nondependent opioid abuse in remission
	191918009	Nondependent cocaine abuse, continuous
	191920007	Nondependent cocaine abuse in remission
	191937000	Nondependent mixed drug abuse, episodic
	191938005	Nondependent mixed drug abuse in remission
	229003004	Cocaine freebase
	231482005	Active drug dependence
	237228001	Pregnancy and drug dependence
	288791000119106	History of cocaine abuse
	288811000119105	History of heroine abuse
	288831000119100	History of opioid abuse
	288851000119106	Opioid-induced mood disorder due to opioid abuse
	292063009	Fentanyl adverse reaction
	295175007	Accidental heroin overdose
	310653000	Drug addiction therapy - methadone
	34150001	Drug dependence in mother complicating pregnancy,
	373492002	childbirth AND/OR puerperium
		Fentanyl
	424848002	Recreational drug user
	425533007	Episodic drug abuse
	428370001	Psychostimulant withdrawal
	429782000	Cocaine misuse
	762327001	Psychotic disorder with delusions caused by stimulant Methamphetamine
	8692006	Heroin overdose of undetermined intent
	295176008	Fentanyl overdose of undetermined intent
	295196000	Drug overdose of undetermined intent
	371341003	Overdose of opiate analgesic of undetermined intent
	295213004	Accidental overdose of opiate
	297199006	
ICD-10	F11	Opioid related disorders
	F14	Cocaine related disorders
	F15	Stimulant (methamphetamine) related disorders
	T40.0	Poisoning by, adverse effect of and underdosing of opium
	T40.1	Poisoning by and adverse effect of heroin
	T40.2	Poisoning by, adverse effect of and underdosing of other opioids
	T40.3	Poisoning by, adverse effect of and underdosing of methadone
	T40.4	

© 2025 Goodman-Meza D et al. *JAMA Network Open*.

Database	Terms/Codes	Descriptors
	T40.5	Poisoning by, adverse effect of and underdosing of other synthetic narcotics
	T40.6	Poisoning by, adverse effect of and underdosing of cocaine
	T43.62	Poisoning by, adverse effect of and underdosing of other and unspecified narcotics
	T43.64	Poisoning by, adverse effect of and underdosing of amphetamines
	T43.65	Poisoning by ecstasy
	T43.66	Poisoning by, adverse effect of and underdosing of methamphetamines
		Poisoning by, adverse effect of and underdosing of other psychostimulants
ICD-9	304.0X	Opioid type dependence
	304.7X	Combinations of opioid type drug with any other drug dependence
	305.5X	Opioid abuse
	E854.2	Poisoning by opiates and related narcotics
		Poisoning by opiate antagonists
		Accidental poisoning by heroin
		Accidental poisoning by methadone
		Accidental poisoning by other opiates and related narcotics
		Adverse effects of opiate antagonists
		Accidental poisoning by psychostimulants
		Poisoning by psychostimulant, unspecified
		Poisoning by amphetamines
		Poisoning by other psychostimulants

© 2025 Goodman-Meza D et al. *JAMA Network Open*.

eTable 6. Antibiotic Characteristics for PWUD Group

Variable	SOC, N = 4,806	laLGP, N = 241
**Terminal antibiotics, n (%)**		
Vancomycin	933 (19%)	
Cefazolin	848 (18%)	
Trimethoprim/sulfamethoxazole	572 (12%)	
Doxycycline	556 (12%)	
Cephalexin	554 (12%)	
Daptomycin	445 (9.3%)	
Linezolid	383 (8.0%)	
Nafcillin	210 (4.4%)	
Cefadroxil	30 (0.6%)	
Oxacillin	92 (1.9%)	
Ceftaroline	86 (1.8%)	
**Days, terminal antibiotics, Median (IQR)**	13 (6 – 30)	
**Days, prior to laLGP, Median (IQR)**		22 (12 – 41)
**laLGP doses, Median (IQR)**		2.00 (1.00 – 2.00)
**Abbreviations: laLGP, long acting lipoglycopeptide; IQR, interquartile range; SOC, standard of care antibiotics.<br>Antibiotics less than 1% were truncated from list.**		

© 2025 Goodman-Meza D et al. *JAMA Network Open*.

eTable 7. Antibiotic Characteristics for Non-PWUD Group

**Variable**	**SOC, N = 36,436**	**laLGP, N = 584**
**Terminal antibiotics, n (%)**		
**Cefazolin**	7,150 (20%)	
**Vancomycin**	6,702 (18%)	
**Cephalexin**	6,409 (18%)	
**TMP/SMX**	4,502 (12%)	
**Doxycycline**	4,251 (12%)	
**Daptomycin**	2,594 (7.1%)	
**Linezolid**	2,105 (5.8%)	
**Nafcillin**	963 (2.6%)	
**Cefadroxil**	476 (1.3%)	
**Oxacillin**	456 (1.3%)	
**Ceftaroline**	376 (1.0%)	
**Days, terminal antibiotics, Median (IQR)**	10 (5 – 27)	
**Days, prior to laLGP, Median (IQR)**		14 (7 – 32)
**laLGP doses, Median (IQR)**		2.00 (1.00 – 2.00)
**Abbreviations: laLGP, long acting lipoglycopeptide; IQR, interquartile range; SOC, standard of care antibiotics. Antibiotics less than 1% were truncated from list.**		

© 2025 Goodman-Meza D et al. *JAMA Network Open*.
