# Supplementary material for: Comparative Effectiveness of Long-Acting Lipoglycopeptides vs Standard-of-Care Antibiotics in Serious Bacterial Infections
Source: JAMA Netw Open. 2025 May 21;8(5):e2511641. doi: 10.1001/jamanetworkopen.2025.11641 (PMC12096263; doi:10.1001/jamanetworkopen.2025.11641)
Supplement: Supplement 2. — Data Sharing Statement [file jamanetwopen-e2511641-s002.pdf]

Data Sharing Statement

Goodman-Meza. Comparative Effectiveness of Long-Acting Lipoglycopeptides vs Standard-of-Care Antibiotics in Serious Bacterial Infections. *JAMA Netw Open*. Published May 21, 2025. doi:10.1001/jamanetworkopen.2025.11641

Data
Data available: No

Additional Information
Explanation for why data not available: Due to contractual agreements with Cerner we are not able to export individual patient data. Researchers could get to the individual patient data but they would have enter into agreement with Cerner and pay a fee for access.
